# Deep learning enhanced terahertz imaging of silkworm eggs development

**DOI:** 10.1016/j.isci.2021.103316

**Published:** 2021-10-19

**Authors:** Hongting Xiong, Jiahua Cai, Weihao Zhang, Jingsheng Hu, Yuexi Deng, Jungang Miao, Zhiyong Tan, Hua Li, Juncheng Cao, Xiaojun Wu

**Affiliations:** 1 School of Electronic and Information Engineering, Beihang University, Beijing 100191, China; 2 School of Cyber Science and Technology, Beihang University, Beijing 100191, China; 3 College of Engineering, Peking University, Beijing 100191, China; 4 Key Laboratory of Terahertz Solid State Technology, Shanghai Institute of Microsystem and Information Technology, Chinese Academy of Sciences, Shanghai 200050 China; 5 Center of Materials Science and Optoelectronics Engineering, University of Chinese Academy of Sciences, Beijing 100049, China; 6 Wuhan National Laboratory for Optoelectronics, Huazhong University of Science and Technology, Wuhan 430074 China

**Keywords:** Wave imaging, Applied physics, Machine learning

## Abstract

Terahertz (THz) technology lays the foundation for next-generation high-speed wireless communication, nondestructive testing, food safety inspecting, and medical applications. When THz technology is integrated by artificial intelligence (AI), it is confidently expected that THz technology could be accelerated from the laboratory research stage to practical industrial applications. Employing THz video imaging, we can gain more insights into the internal morphology of silkworm egg. Deep learning algorithm combined with THz silkworm egg images, rapid recognition of the silkworm egg development stages is successfully demonstrated, with a recognition accuracy of ∼98.5%. Through the fusion of optical imaging and THz imaging, we further improve the AI recognition accuracy of silkworm egg development stages to ∼99.2%. The proposed THz imaging technology not only features the intrinsic THz imaging advantages, but also possesses AI merits of low time consuming and high recognition accuracy, which can be extended to other application scenarios.

## Highlights

• THz QCL video system recorded the changing of the silkworm egg internal morphology
• Established a silkworm egg dataset corresponding to THz image and optical image
• Combining deep learning and data fusion to detect silkworm egg development stages

## Graphical Abstract

**Figure:**
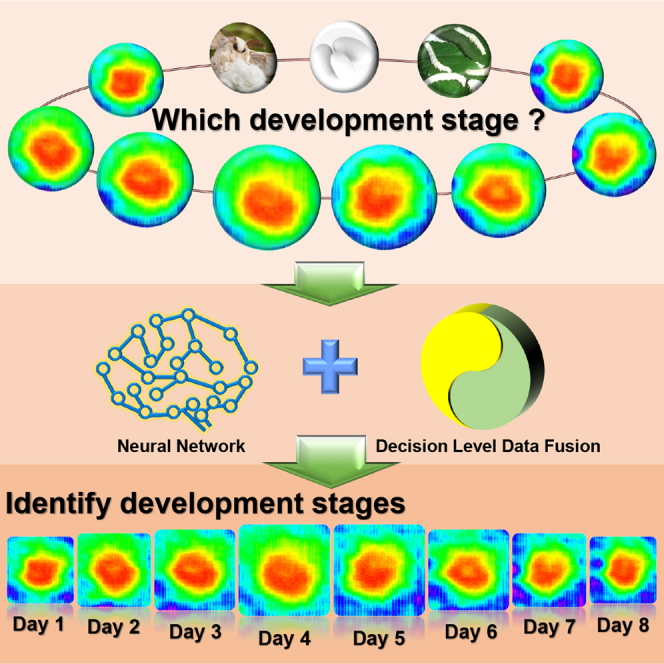

## Teaser

Terahertz imaging; Applied physics; Machine learning

## Introduction

Terahertz (THz, 0.1–10 THz) waves, located between microwave and near-infrared light, featured many distinguishing properties, and have been recognized as the basis of next-generation high-speed wireless communication and one of the most powerful non-invasive spectral sensing and imaging techniques (Ma et al., 2018; Sengupta et al., 2018; Venkatesh et al., 2020; Yang et al., 2020). THz radiation is non-ionizing and water sensitive, which will not cause ionizing damage to biomaterial tissues and promote the recognition application for biological samples. However, most THz imaging techniques based on scanning method are still suffering the time-consuming disadvantage, greatly hindering the widespread applications of THz technology (Cocker et al., 2013; Watts et al., 2014). Along with the accelerated advances of high-power THz sources (Chen et al., 2019; Khalatpour et al., 2021; Samizadeh Nikoo et al., 2020; Zhang et al., 2021; Zhao et al., 2020) and various high-sensitivity THz detectors (Gayduchenko et al., 2021; Guo et al., 2020; Ma et al., 2019; Peng et al., 2020), THz spectral imaging technology turns out to be feasible. Among these devices, high-power THz quantum cascaded laser (QCL) light sources are facing the dawn of moving from the laboratory research stage to practical applications (Khalatpour et al., 2021; Stantchey et al., 2020), and THz video imaging technique is expected to usher in rapid development (Zhou et al., 2018). One of the most advantages of THz video imaging lies in quickly obtaining sample images, laying a solid foundation for the subsequent THz image processing. Although recent advances in deep learning have been providing various versatile solutions for optics (Fan et al., 2020; Norris et al., 2020; Varadarajan et al., 2020), inspiring the intersection of deep learning and THz technology (Veli et al., 2021), the intelligent THz imaging technology and its applications are still in their infancy, and further explorations are highly demanded.

**Figure 1:**
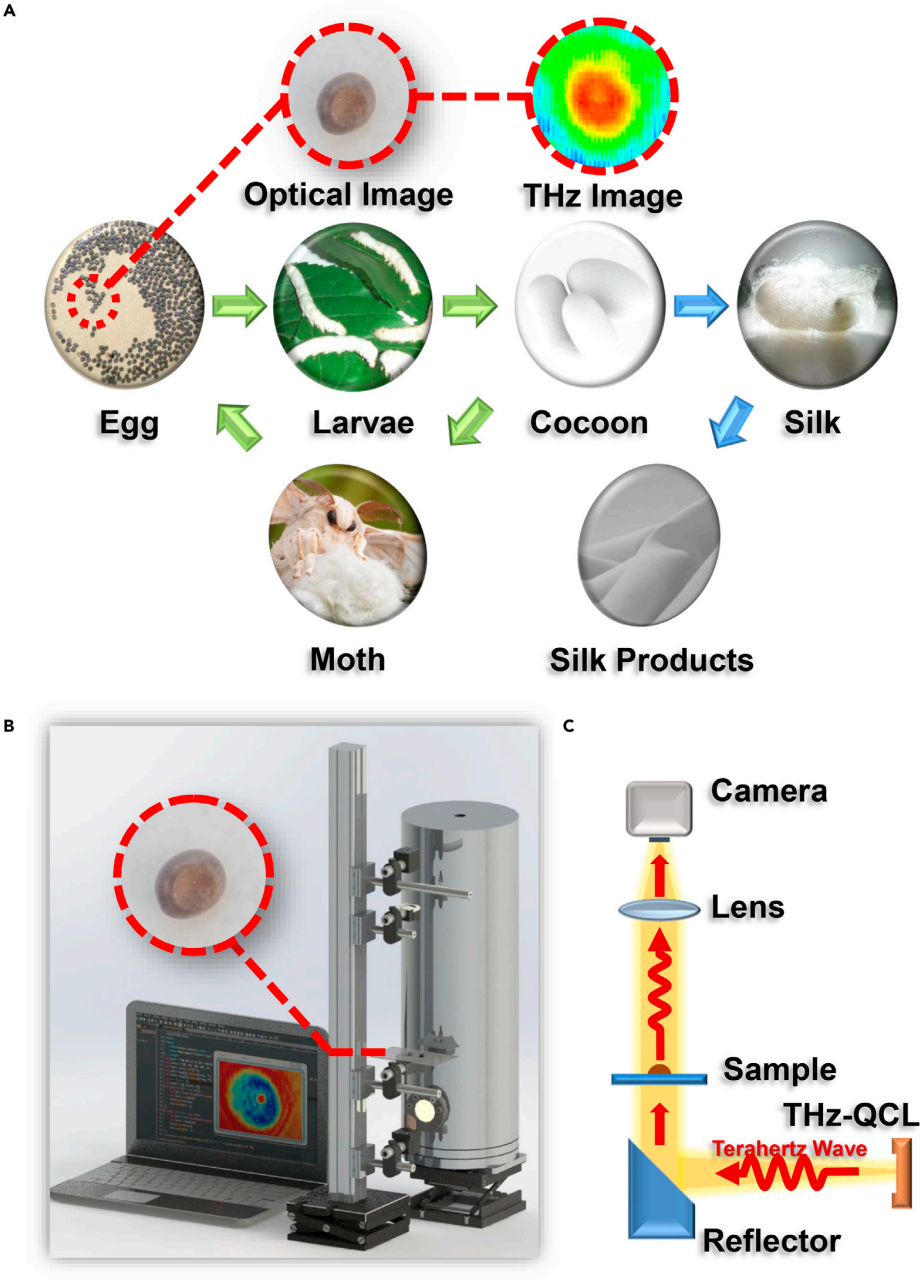
Schematic diagram of the silk industry and THz QCL video imaging system (A) The life of a silkworm goes through 4 stages, namely silkworm eggs, larvae, cocoons, and moths, among which the silkworm egg development plays significant roles in the whole life cycle. After silkworm eggs are cocooned, silk can be obtained by spinning. Using silk, we can make many kinds of silk products. (B) THz QCL video imaging system primary includes a liquid nitrogen cryogenically cooled QCL chip delivering 4.3 THz wavelength, a 90° off-axis parabolic mirror (focal length = 50.8 mm), a high-resistivity silicon lens (focal length = 50 mm), and a THz camera array with a video imaging frame rate up to 25 frames. (C) THz imaging optical path. The emitted THz waves are first collimated by the parabolic mirror, resulting in an effective imaging field diameter of ∼10 mm. The developing silkworm egg is placed onto a plastic sample holder, and the transmitted THz waves are focused by the silicon lens onto the camera array.

One possible application could be in multifunctional silk biomaterials and silk optics, such as monitoring silkworm egg development and silk spinning in the cocoon. Recent emergent silk biomaterials have gained rapid progress due to their unique properties of toughness, elasticity, and lightweight (Cai et al., 2020; Lee et al., 2020; Sun et al., 2020). Among them, silkworm silk is one of the most intensively investigated and practically applied (Min et al., 2017; Wang et al., 2017, Wang et al., 2019b). Silk is not only used for clothing production but also served as multifunctional flexible protein materials, such as coating materials that are thermal regulation applications similar to "smart windows" (Wang et al., 2019a), which have excellent electromagnetic interference shielding, super-hydrophobicity, and highly sensitive humidity response (Liu et al., 2019). However, perhaps one of the most effective ways to realize flexible control of the functional silk biomaterials was to start with sericulture (Wang et al., 2016). The process of sericulture can be divided into many stages, as exhibited in Figure 1A. Automatic detection of cocoon gender classification has been realized (Raj et al., 2019). However, silkworm eggs play an important role in the whole sericulture industry chain, and automatic detection has not been realized. During this period, physiological processes such as sex determination, tissue organ formation and differentiation, diapause, and pigmentation occur (Xia et al., 2014). Therefore, effectively monitoring silkworm egg development is essential for the whole chain process of sericulture. Fine breeding is helpful to obtain high-efficiency silk production, which makes monitoring silkworm egg development valuable. Traditional monitoring approaches for silkworm egg development can be divided into invasive (anatomy) and non-invasive (spectroscopy) methods (Qiu et al., 2021). Anatomy technology will damage the silkworm egg tissue and affect its further development, whereas traditional spectroscopy methods suffer both the limited spectral information for the specific areas of silkworm egg and established model accuracy. Therefore, applying a non-invasive, fast and accurate THz imaging technique to monitoring silkworm egg development is valuable and has not yet been reported.

Here we employ a THz QCL real-time video imaging system working at 4.3 THz to systematically investigate the silkworm egg development process. Combined with the deep learning algorithm, we have successfully achieved the non-destructive recognition of the silkworm egg development stage with very high accuracy. Fused with optical imaging, the accuracy is further enhanced. We believe this demonstrated THz technology can be extended to the recognition of other biological samples, thereby accelerating the real application of THz technology in industry, agriculture, and biomedicine.

## Results and discussion

### Sample preparation and experimental setup

Moth lay diapause egg in an unsuitable circumstance with non-appropriate ambient temperature and insufficient food during the spawning period. The silkworm egg measured in our experiments were diapause egg and directly purchased. The silkworm breed used in our experiment is Bombyx mori Linnaeus, which originated in China. We put the diapause eggs in an ambient temperature with 20–22°C temperature, and they resumed development. The THz video imaging system, as depicted in Figure 1B, includes a QCL laser with center frequency 4.3 THz, a liquid nitrogen cryogenically cooling system, a THz camera array, and several THz optics. The THz imaging optical path is exhibited in Figure 1C. The radiated THz wave from the QCL chip is first collimated by a 90° off-axis parabolic mirror (focal length = 50.8 mm), then passes through a sample holder, and finally is focused onto the camera array via a high-resistively silicon lens (focal length = 50 mm). The QCL video acquisition system has adjusted the light path before the silkworm egg image acquisition. During the entire data acquisition process, the positions of the camera, lens, sample holder, and QCL source are fixed. The imaging frame rate is 9 fps, the spatial resolution is ∼300 μm, and the diameter of effective imaging area is ∼10 mm. The length of the optical path exposed to the air is ∼31.2 cm. Figure S1 illustrated the stability of THz light spot.

The tested silkworm egg was placed on the sample holder in the collimated THz beam. Figure S2 exhibits the attenuation of the THz wave by the sample holder. Only one silkworm egg was put in each measurement. To get enough data for deep learning, 145 diapause egg during the development process were measured at a fixed time every day via the THz QCL imaging system, and 11,680 images for 8 days were obtained. All these measurements were conducted at a constant temperature of ∼22°C and relative humidity ∼40%, and the THz system was not purged or pumped.

### Silkworm egg internal THz morphology

**Figure 2:**
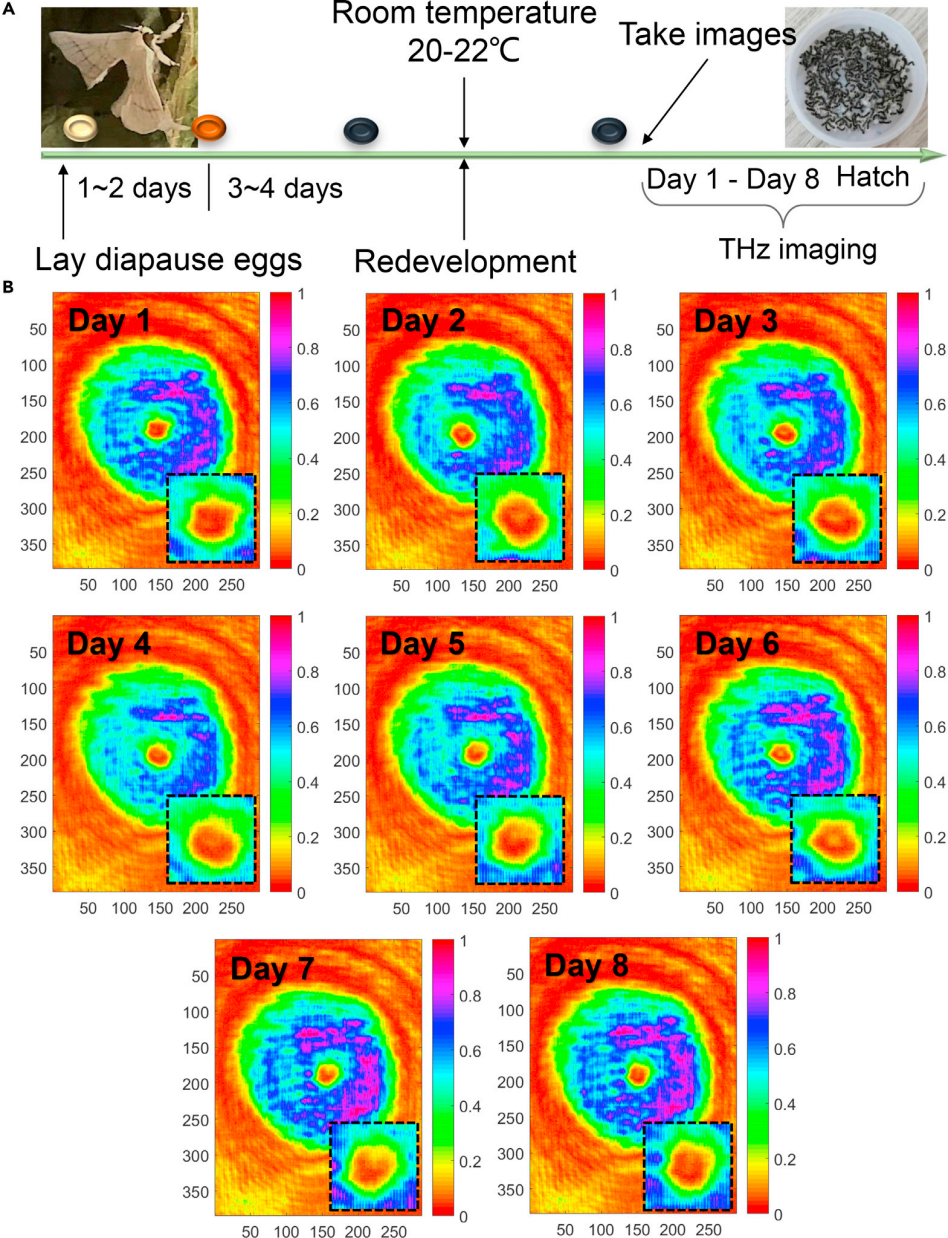
Silkworm egg internal morphology captured by THz video imaging (A) The specific time of our research. (B) In the early development stages of Day 1, the THz images appear as uniform distribution, whereas Day 2 to Day 6 present ring shapes, implying the forming dawn of ant silkworms. Day 7 and Day 8 give split ring shaping, and on Day 8 you can see a deep U-shape, which means it has formed ant silkworm, predicting the ant silkworm will break its shell soon.

The silkworm eggs just laid by the silkworm moths are light yellow, which turns reddish-brown after 1–2 days and dark blue after 3–4 days. At this time, the silkworm eggs stop developing until the temperature returns to about 20°C to regenerate. Figure 2A shows that the specific time we are interested in is 8 days before the birth of the ant silkworm. All the data we used are within 8 days before ant silkworms breaking the shells. Here we define Day 1 as 8 days before hatching, hence Day 8 is for less than 24 h before hatching. When collecting images of silkworm eggs, there is a "+" marker in the imaging software to ensure that the silkworm egg is placed in the center area of the THz light spot. With this method, we can safely solve the alignment issue. Figure 2B illustrates typical THz images of the developing silkworm egg within 8 days. As a quick glance, we can distinguish the silkworm egg in the THz light spot for each image, presenting as red round spots in the image central areas. This phenomenon implies strong absorption between THz waves and silkworm egg, because the internal proteins, water, and inorganic salt are heavily absorbing. Figure S3 shows silkworm egg shells are highly transparent for THz waves. We analyze the THz images of 145 silkworm egg and find that the internal morphology changes of silkworm eggs are roughly divided into three stages: uniform distribution, ring shapes, and split ring shaping during the development process. For example, the silkworm eggs shown in Figure 2B, on Day 1 the silkworm egg internal THz morphologies exhibit uniform distribution, indicating silkworm life being developing from intangible to tangible. Day 3 can be treated as a shaping threshold, as a ring can be preliminarily recorded, but it becomes more obvious in the THz image of day 6. It can be safely estimated that an ant silkworm is formed because a split ring shape is successfully captured by THz imaging on Day 7. On Day 8, you can find a deep U-shape in the middle of the silkworm egg, which is the ant silkworm that is about to break out of its shell. After the eighth day, the ant silkworm crawls out of the egg shell. These experimental results conclusively and evidently indicate that THz imaging can be successfully applied to monitor the silkworm egg development process. Video S1 dynamic displays the internal morphology of silkworm eggs at different development stages. From these figures we can see that THz image of Day 1 shows uniform distribution and Day 6 exhibits ring shape, whereas that for Day 7 appears very irregular behavior. We can further explicitly corroborate the validity of THz imaging for capturing silkworm egg internal morphology and their general development process. The process of monitoring the development stages of silkworm egg by THz imaging technology demonstrates the feasibility of "seeing" the life origin, that is, the development of amorphous proteins and inorganic salts into movable insects. Besides, with our proposed method, we can also monitor the silkworm eggs for 2–3 days. If the internal morphology varied, it meant the silkworm eggs had the capability to hatch. On the contrary, the silkworm egg was dead.

Video S1. Internal morphology of silkworm eggs at different development stages, related to Figure 2

### Intelligent recognition of THz images

**Figure 3:**
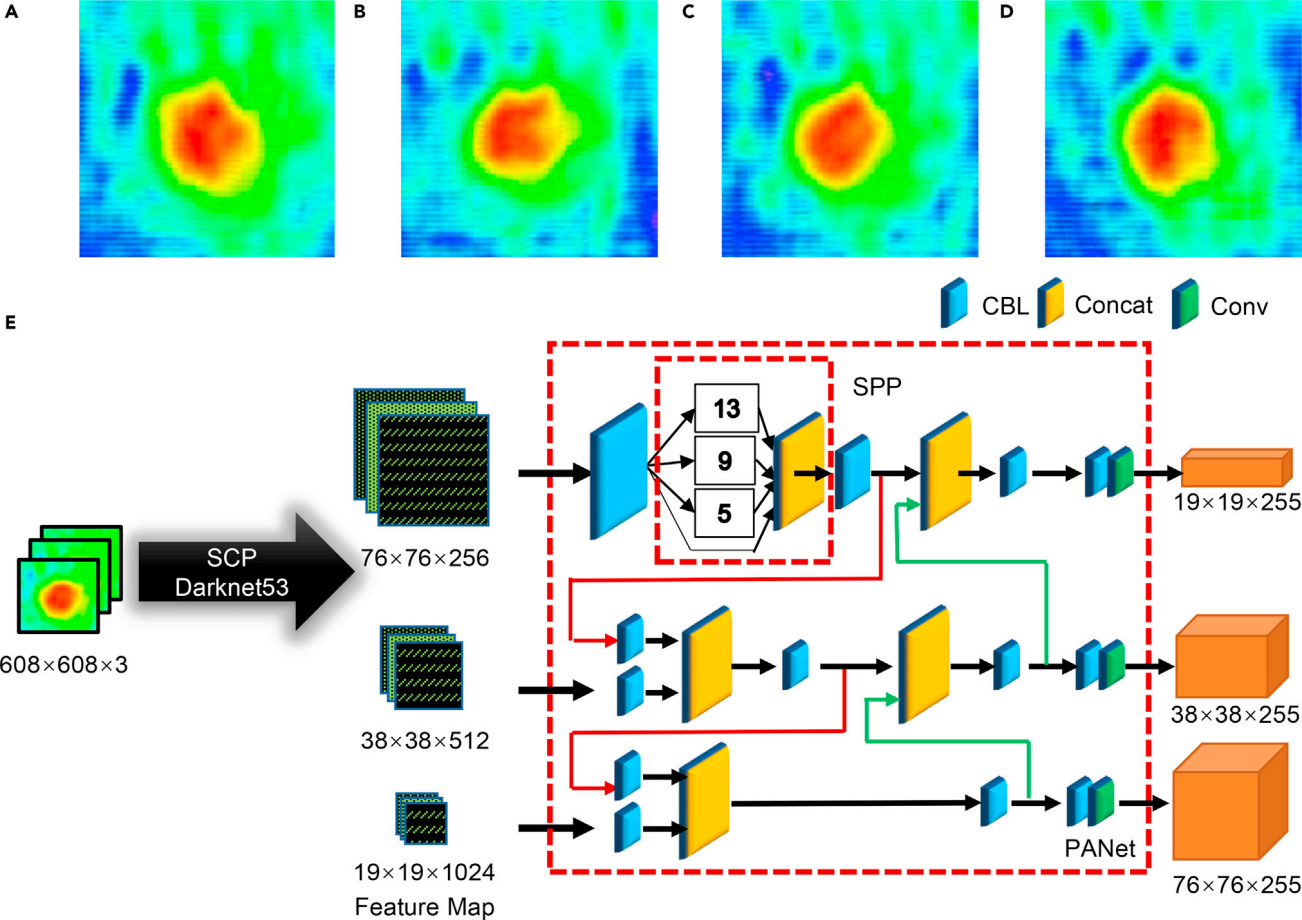
Artificial intelligence THz recognition of silkworm egg development stages (A–D) THz images of 4 silkworm eggs with the same development stages. We cannot make the decision on which stage they belong to, resulting in fine judgment difficulty. (E) Deep learning network architecture. The arrow represents the information transfer process, the red arrow represents up-sampling, and the green arrow represents down-sampling.

Although we can approximately estimate the silkworm development stage in the egg via THz video imaging, it is still very challenging to distinguish THz images manually for faster and more accurate recognition. Generally speaking, silkworm egg featuring individual differences may present various THz morphology even at the same development stages. Taking Figures 3A–3D as an example, from these images, we cannot judge which stage these eggs belong to. However, when AI technology is introduced into THz image processing, we can get the silkworm egg development stage, based on the convolutional neural network (CNN) model trained by a large number of prelabeled data.

Among various CNN models, You Only Look Once (YOLO) network architecture was first proposed in 2016, creating an unprecedented one-stage target recognition method (Redmon et al., 2016). The proposed-free idea can greatly improve the speed of target recognition and realize real-time recognition with high frame rate. Hence, we employ the YOLO v4 neural network architecture (Bochkovskiy et al., 2020), given in Figure 3E, to enhance the THz recognition accuracy. The experimental environment of this paper is under Window10 system, GPU is TITAN RTX, AMD RYZEN Threadripper 3960X CPU @3.8GHz, 2 T hard disk, 128 G memory. First, we divide the silkworm egg images into 608 × 608 grids to improve recognition accuracy. Afterward, the feature map of silkworm egg including local and global features are extracted via a convolution network spatial-channel parallelism darknet 53 (SCPDarknet 53) (Fan et al., 2019), forcing the local feature to be focused onto specific global features. Figure S4A presents the SCPDarknet 53 structure. The loss of the spatial dimension-channel dimension parallel mechanism, defined as $L_{SCP}$, is as follows:

(Equation 1) $$L_{SCP}=\sum_{r=1}^{R}{\left\|f_{s,r}-f_{c,r}\right\|}_{2}^{2}$$

where $f_{s,r}$ is the r^th^ local feature, and $f_{c,r}$ is the r^th^ part of the global feature. Then, we use the feature maps of 76 × 76, 38 × 38, and 19 × 19 scales to realize silkworm egg image recognition. Input different scale feature maps to the spatial pyramid pooling (SPP) network (He et al., 2015), we obtain pooling features of the same length, and use the padding method to keep a constant size of the output feature map. In the feature pyramid image, the bottom part contains the local information of the silkworm egg image, whereas the top part contains semantic information. By employing the path aggregation network (PANet) (Liu et al., 2018), the bottom-up path realizes the concatenation of two kinds of information. Consequently, the silkworm egg development stage is successfully identified. We use the cluster method to recalculate the width and height of the anchor box of the dataset, which are (78, 59), (89, 57), (83, 66), (94, 62), (92, 68), (106, 65), (92, 76), (102, 71), and (109, 78). The number of training epochs is 16,000, and the training period is ∼35.24 h. Figures S5 and S6 show the training loss curves before and after optimization.

**Figure 4:**
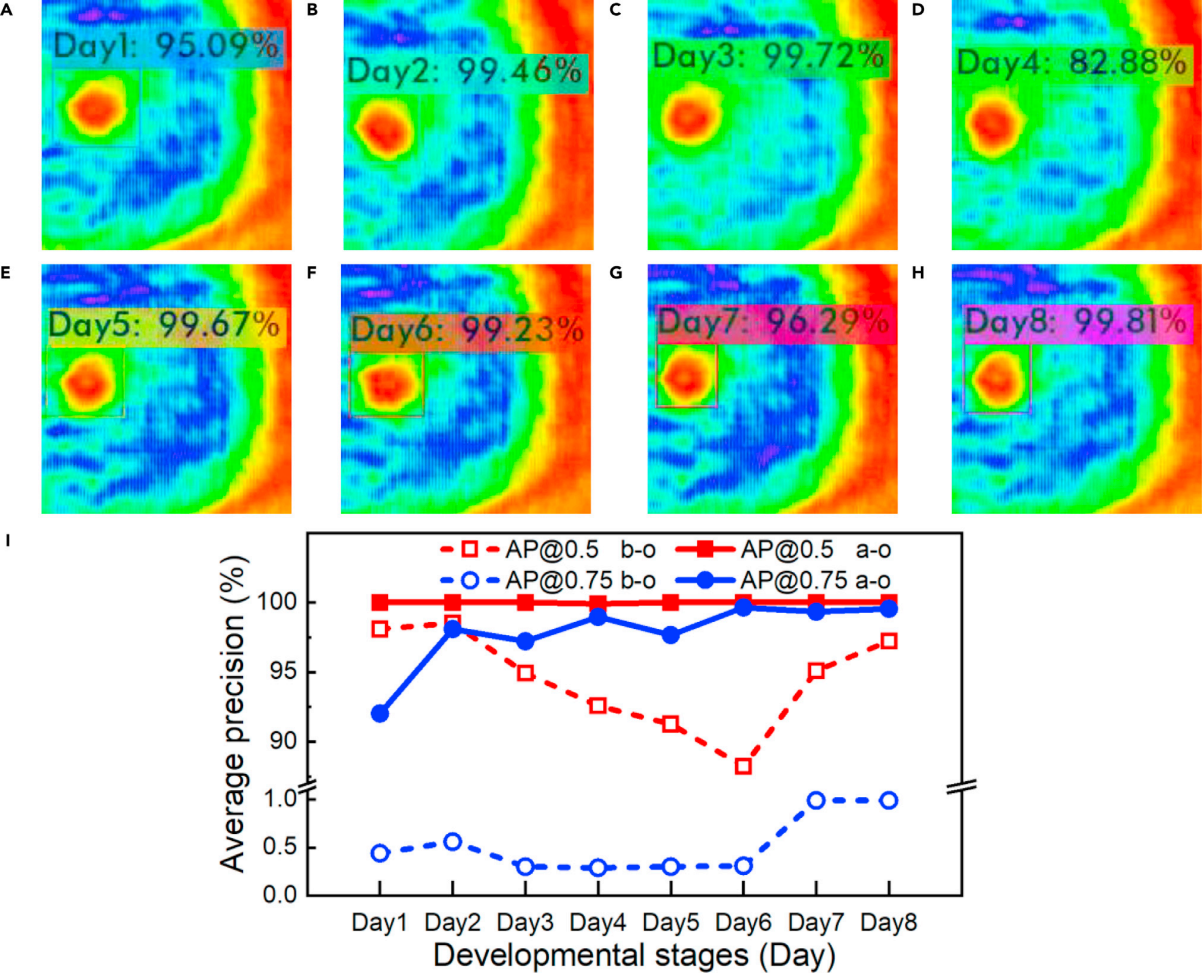
Experimental results of AI THz recognition on single silkworm egg (A–H) Intelligent THz recognition results of different development stages of a specific silkworm egg. (I) Average precision of deep learning network for every category. The dashed and solid lines represent the precision before and after optimization, respectively. The red and blue lines represent the precision of *IoU* more than 0.5 and 0.75, respectively.

**Figure 5:**
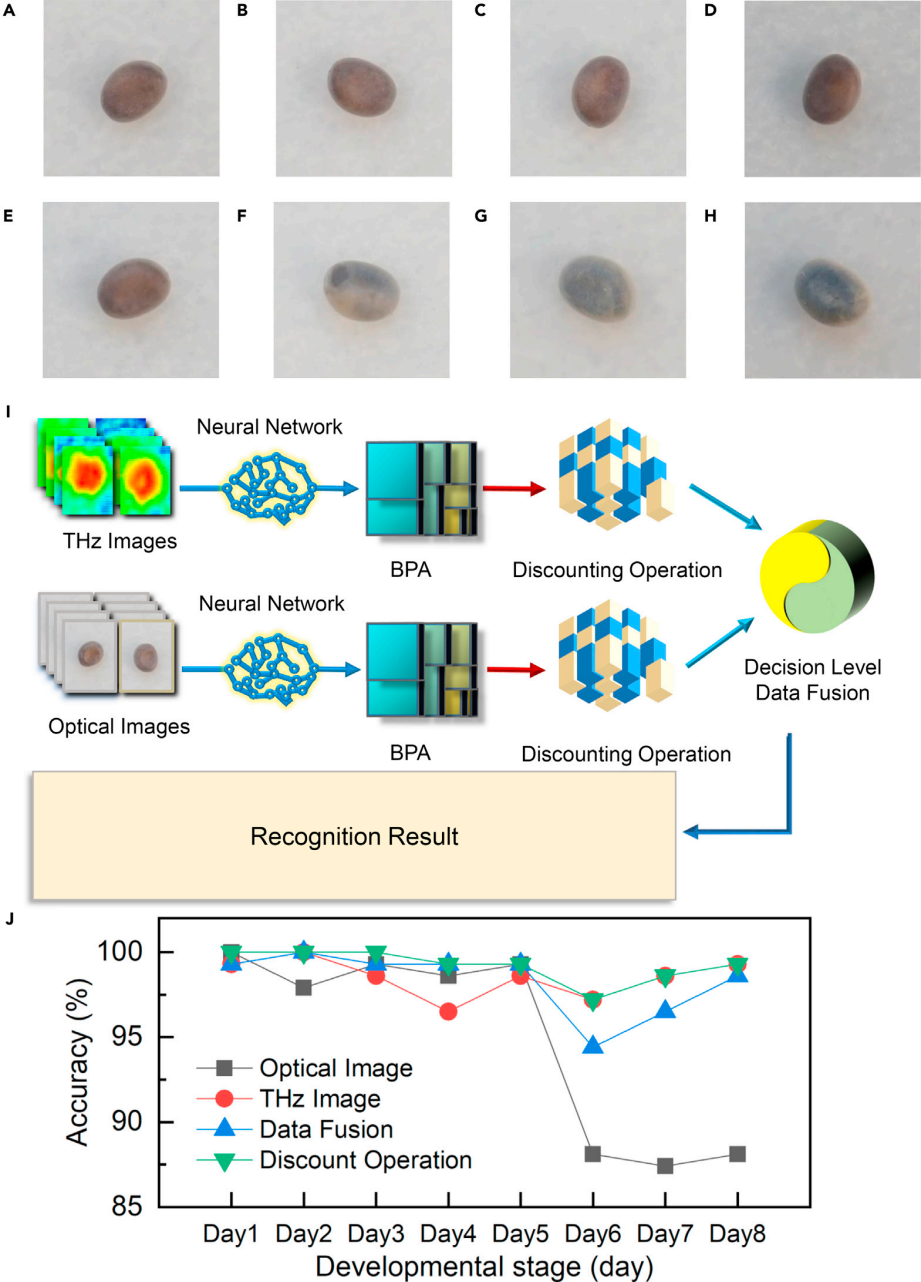
Data fusion of THz and optical imaging (A–H) Optical image of a specific silkworm egg continuously developing from Day 1 to Day 8, respectively. (I) Fusion recognition process of THz imaging and optical imaging. (J) Recognition accuracy results of silkworm egg development with optical imaging, THz imaging, data fusion without and with discount operation. Optical imaging data fusion can, to some extent, improve THz intelligent recognition for the first five days. However, for the latter three days of the silkworm egg development process, THz imaging is the unprecedented powerful technique to judge the development stage.

Figure 4 depicts random experimental results of AI recognition for one silkworm egg. For the silkworm egg being continuously developed for 8 days, AI THz imaging technology can accurately judge the development stage within 300 ms. As shown in Figures 4A–4H, we can accurately locate the position of the silkworm egg in THz images (see the square in each image) and then give the development days. A dynamic recognition video can be found in Video S2. For this silkworm egg, the confidence rate of Day 2, 3, 5, 6, and 8 is almost close to 100%, which shows that the model we have established is highly reliable in distinguishing the developmental stages of silkworm eggs. Figures 4A–4H shows that the confidence rate on Day 1 and Day 7 is also higher than 95%, and on Day 4 it is slightly lower but reaches 80%. Figure 4I plots the average precision (*AP*) of the validation set before and after model optimization. When the intersection over union (*IoU*) threshold is set to 0.5 and 0.75, the mean average precision (*mAP*) before optimization is 94.49% and 0.52%. However, the *mAP* after optimization is increased to 99.99% and 97.81%, respectively. After optimization, when the *IoU* threshold is 0.75, the *AP* of Day1 to Day8 is increased to 92.03%, 98.08%, 97.21%, 98.96%, 97.66%, 99.64%, 99.33%, and 99.54%, respectively. We use 1,144 THz images that have not been trained to test the generalization ability of the model. Figure 5J exhibits that the recognition accuracy of Day 1 to Day 8 is 99.3%, 100%, 98.60%, 96.50%, 98.6%, 97.20%, 98.60%, and 99.30%, respectively. Although the development recognition is close to 100%, the system is not overfitting. To avoid this, we enhanced the image of the input network by using CutMix, Mosaic, and self-adversarial training. Then we employed a label smoothing regularization method to make the cluster between categories more compact. Finally, we used Dropblock method to force the network to learn other regional information, which is more conducive to extracting global features. These results further verify that the AI THz recognition technology can not only improve the accuracy but also save manual labor and time consumption.

Video S2. Real-time detection results of silkworm egg development stage, related to Figure 4

### Multisource data fusion between THz and optical images

Up to now, THz recognition of silkworm egg development stage based on YOLO v4 has been already successfully achieved. How to further improve the accuracy is under further investigation in this section. To this end, a multisource data fusion method based on Dempster-Shafer's (D-S) evidence theory (Dempster, 1967) is employed to fuse the recognition results of THz imaging datasets and optical imaging datasets (Xiao, 2019). For this experiment, we also choose a silkworm egg that develops continuously for 8 days to record its optical and THz images. Typical optical images from Day 1 to Day 8 are exhibited in Figures 5A–5H, respectively. From Figures 5A–5E, we can see that the silkworm egg color does not vary significantly from Day 1 to Day 5. However, on Day 6, the silkworm egg shell begins to become a little translucent, and pigment deposits start to appear inside the silkworm egg as shown in Figure 5F. Subsequently, the whole egg shell gradually becomes translucent as shown in Figures 5G and 5H, and the color inside the shell turns blue and dark until the hatching of the ant silkworm is complete.

The recognition result of optical imaging is shown in Figure 5J with a gray line. The first five days of silkworm egg development stages can be well identified by optical images; the accuracy from Day 1 to Day 5 are 100%, 97.90%, 99.30%, 98.60%, and 99.30%, respectively. However, for the latter three days, the recognition accuracy is abruptly reduced, which are 88.11%, 87.41%, and 88.11% from Day 6 to Day 8, respectively. If the color variation was evident for the whole development process, the proposed model could be employed to further improve the recognition accuracy.

From these experimental results, the recognition accuracy of optical imaging in the first five days is slightly higher than that of THz imaging, whereas it becomes much lower than that of THz imaging in the next three days. We analyzed the results of the last three days and found that for the recognition results of the last three days, more than 88.4% of the false recognition was identified within the range of the last three days. Therefore, although the optical appearance of silkworm eggs in the last three days is significantly different from that in the first five days, the changes are not obvious between the last three days. The recognition results in the last three days are easily confused with each other. To further improve the recognition accuracy, we fuse the two kinds of data. Fusion recognition process of THz imaging and optical imaging is illustrated in Figure 5I. THz images and optical images are subjected to YOLO v4 neural networks to extract features and obtain their identification results, and the respective basic probability assignment (BPA) is then combined using the D-S theory. If necessary, the BPA can also be discounted. See Supplementary information S6 for basic theories of D-S theory and discount operation (Mercier et al., 2008).

The recognition result after data fusion process is shown in Figure 5J with a blue line. The accuracy from Day 1 to Day 8 are 99.30%, 100%, 99.30%, 99.30%, 99.30%, 94.41%, 96.50%, and 98.60%, respectively. Compared with the recognition results of THz images, the recognition accuracy increased from Day 3 to Day 5 and decreased from Day 6 to Day8. The recognition accuracy of optical images is higher in the first five days, which is beneficial to improve the accuracy after data fusion process, whereas the accuracy of optical images is lower in the next three days, which will have a negative impact on the data fusion process. Therefore, a discount factor *α* = 0.005 is set for the recognition results of THz images for the first five days, and another discount factor *α* = 0.15 is set for the recognition results for the last three days of the optical images. The recognition result after data fusion process and discount operation is shown in Figure 5J with a green line. The accuracy from Day 1 to Day 8 are 100%, 100%, 100%, 99.30%, 99.30%, 97.20%, 98.60%, and 99.30%, respectively. Compared with the recognition results of THz images, the average recognition accuracy of 8 days increased 0.7%. Through the fusion of optical imaging and THz imaging technology in the silkworm egg development stage, the accuracy of the first 5 days can be effectively improved. Due to the low recognition accuracy of optical images for the latter three days, using data fusion is still difficult to improve the final accuracy, while maintaining the recognition accuracy of THz imaging, further manifesting the convenient application feasibility of THz recognition of silkworm egg development.

### Conclusions

In summary, we apply THz imaging technology, for the first time, to successfully unveil the internal morphology of silkworm egg at different development stages, and established a one-to-one corresponding THz image and optical image silkworm egg development dataset. By analyzing the THz sequential images of 145 silkworm eggs, we clarify that the morphology variation obeys a tendency of a uniform distribution to a ring shape, and finally split ring shaping along with the development stages. To improve the recognition accuracy and shorten the recognition time, AI method named YOLO v4 architecture is introduced, and a 98.5% recognition accuracy is achieved. Fusion of internal THz images and external optical images of silkworm egg at the decision-making level further improves the recognition accuracy to 99.2%. Our proposed “pregnancy test of silkworm egg” with THz imaging technique may be valuable for silk optics and inspirational for the AI accelerated THz technology and applications.

### Limitations of the study

We only used one kind of silkworm as the experimental sample, lacking the experimental data of other silkworm eggs before hatching. When establishing the dataset, only the experimental data of 8 days before silkworm egg hatching were selected, and the longer-term development days could not be judged.

## STAR★Methods

### Key resources table

**Table:** 

REAGENT or RESOURCE	SOURCE	IDENTIFIER
**Software and algorithms**		

Silkworm egg development model	This paper	https://zenodo.org/record/5556786#.YWBbrdpBx3g https://doi.org/10.5281/zenodo.5556786
YOLO v4	Bochkovskiy et al., 2020	https://arxiv.org/pdf/2004.10934.pdf
Visual Studio 2019	Microsoft corporate	https://visualstudio.microsoft.com/zh-hans/vs/

**Other**		

Silkworm egg dataset	This paper	https://zenodo.org/record/5557107#.YWBcDNpBx3g https://doi.org/10.5281/zenodo.5557107

### Resource availability

#### Lead contact

Further information and requests for resources should be directed to and will be fulfilled by the lead contact, Xiaojun Wu (xiaojunwu@buaa.edu.cn).

#### Material availability

The study did not generate any unique reagents.

### Method details

#### THz QCL video imaging system

The THz QCL video imaging system is mainly composed of a QCL source at 4.3 THz and a THz camera array (resolution of 300 μm). The THz wave radiated from the source passes through an off-axis parabolic mirror (focal length = 50.8 mm), then passes through the silkworm egg samples, and then is focused onto the imaging array of the THz camera using a high resistivity silicon lens (focal length = 50 mm). We can get both static images and dynamic THz video with this system because of its high frame rate of 25 fps (in this experiment, we set the frame rate is 9 fps).

#### Silkworm egg dataset

The experimental samples are 145 silkworm eggs in the diapause period and placed in a suitable temperature and humidity environment to ensure that the temperature and humidity of each silkworm egg during incubation are consistent. Other interference factors except individual differences are excluded. The THz radiation intensity received by the THz camera can reflect the internal information of silkworm egg. There are some preprocessing applied before feeding the images to deep learning model. The method for image preprocessing includes three steps: (Ⅰ) Normalize the intensity information of the acquired THz radiation; (Ⅱ) Rearranged it according to 384 × 288 pixels to obtain the THz grayscale images; (Ⅲ) Add pseudo colors to grayscale images based on the hue saturation value (HSV) to improve the accuracy of AI recognition. We selected the data of each silkworm egg 8 days before hatching, 10 frames of images per day, adding up to 11680 images, the training set and the validation set are divided randomly, with a ratio of 3:1. The same number of optical images are taken at the same time. The optical images are taken by a Sony ILCE-7M2 camera, and the aperture is set to be f/2.8. The shutter speed is set to 1/50 s, and the ISO speed is set to 800. The focal length of the lens is 50 mm. Each silkworm egg is photographed 10 times a day for more than 9 consecutive days, summing more than 15000 images.

#### Deep learning model

The backbone structure of YOLO v4 is DSPDarknet53, which uses the mish function to activate the convolutional layer. The mish function optimizes the negative value truncation problem of the Relu function, and ensures that a small negative gradient flows into the information flow. Meanwhile, the mish function has no borders and avoids the gradient saturation problem. The SPP network is proposed in the Faster-RCNN network architecture, which solves the problem of non-uniform feature map size. However, the improvement in the YOLO architecture is to use padding method during the pooling process to ensure that the output feature map size is fixed. In our case, the SPP structure activation function of the convolutional layer is Leaky Relu, the pooling kernel size is 13, 9, 5, 1, and the pooling step size is 1. We concatenate the feature maps of 4 scales, and the dimension of the final feature map is 19 × 19×2048. When the PANet structure is used to transfer the feature map information, the method of concatenated is adopted to ensure the accuracy of information transfer to the greatest extent. K-means clustering algorithm is used to obtain anchor box size suitable for the silkworm egg dataset(Redmon and Farhadi, 2018), which further improves the bounding box position accuracy. In the experiment, we repeated clustering to obtain 5 sets of anchor box, and calculated the average *IoU*. Table S1 gives all the anchor box sizes base on the cluster method. We choose the anchor box size corresponding to the largest average *IoU*. The loss function of YOLO v4 consists box location loss, confidence loss, and classification loss. We use generalized intersection over union (*GIoU*) to calculate the box location loss(Rezatofighi et al., 2019), and introduce weighting factors to adjust the proportions of the three loss functions.

#### Data fusion

In the prediction bounding box of a single image after non-maximum suppression, the confidence rates of all categories (days) are retained and output to calculate their basic probability assignment. Other prediction bounding boxes are discarded. The confidence rate determined by the trained neural network is denoted as $f_{i}\left(x\right)$ , *i* = 1, 2, 3, 4, 5, 6, 7, 8 is the day number and the corresponding basic probability assignment, defined as *m*(*h*_*i*_), is calculated as follows:

(Equation 2) $$m\left(h_{i}\right)=1-\exp\left(-f_{i}\left(x\right)\right)$$

The basic probability assignment of uncertainty, defined as m(Θ) can be written as:

(Equation 3) $$m\left(\Theta \right)=1-\sum_{i=1}^{N}m\left(h_{i}\right)\text{,}\;N=8$$

After basic probability assignment calculation is completed, decision level data fusion based on Dempster-Shafer's evidence theory is performed.

## Data Availability

• Silkworm eggs data have been deposited at Zenodo and is publicly available as of the date of publication. DOIs are listed in the key resources table. All data reported in this paper will be shared by the lead contact upon request. • All original code has been deposited at Zenodo and is publicly available as of the date of publication. DOIs are listed in the key resources table. • Any additional information required to reanalyze the data reported in this paper is available from the lead contact upon request.
