# Supplementary material for: Deep learning enhanced terahertz imaging of silkworm eggs development
Source: iScience. 2021 Oct 19;24(11):103316. doi: 10.1016/j.isci.2021.103316 (PMC8577140; doi:10.1016/j.isci.2021.103316)
Supplement: Document S1. Figures S1–S6 and Table S1 [file mmc1.pdf]

iScience, Volume *24*

Supplemental information

Deep learning enhanced terahertz imaging of silkworm eggs development

Hongting Xiong, Jiahua Cai, Weihao Zhang, Jingsheng Hu, Yuexi Deng, Jungang Miao, Zhiyong Tan, Hua Li, Juncheng Cao, and Xiaojun Wu

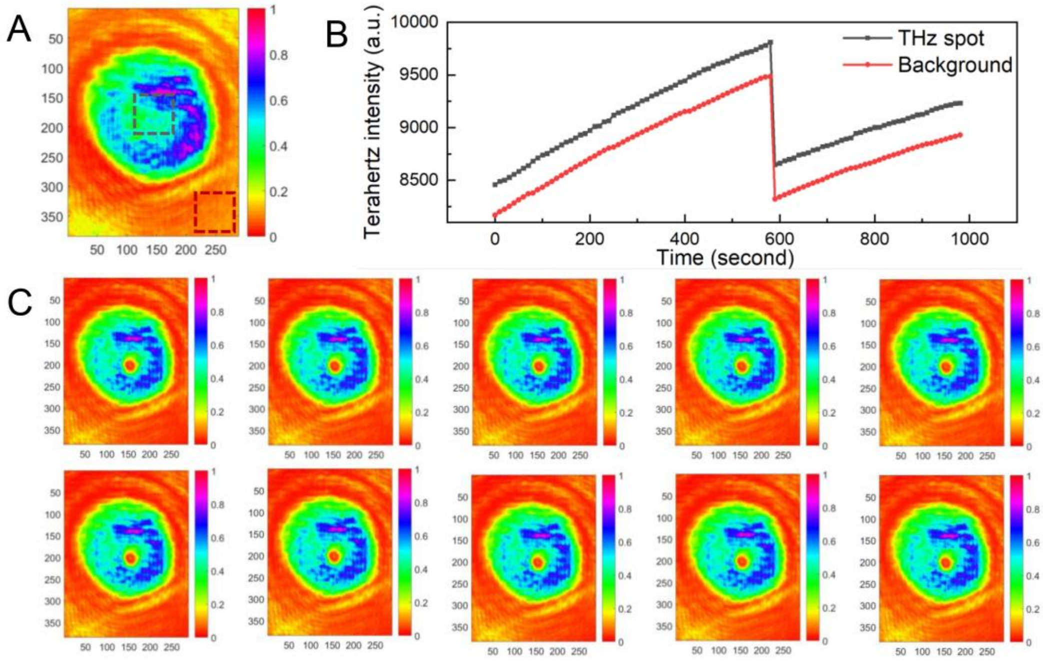

Figure S1. Stability of THz light source, Related to Figure 1. (A) Schematic diagram of camera intensity acquisition location. (B) Raw data obtained by the camera. (C) THz silkworm egg sequential images.

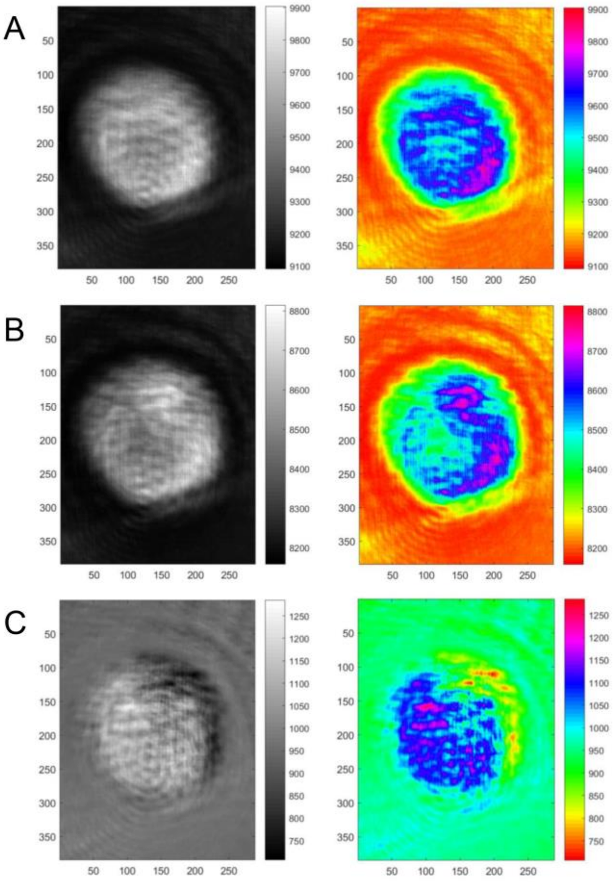

Figure S2. THz spots, Related to Figure 1. THz spot (A) without and (B) with sample holder. (C) THz intensity imaging of sample holder loss.

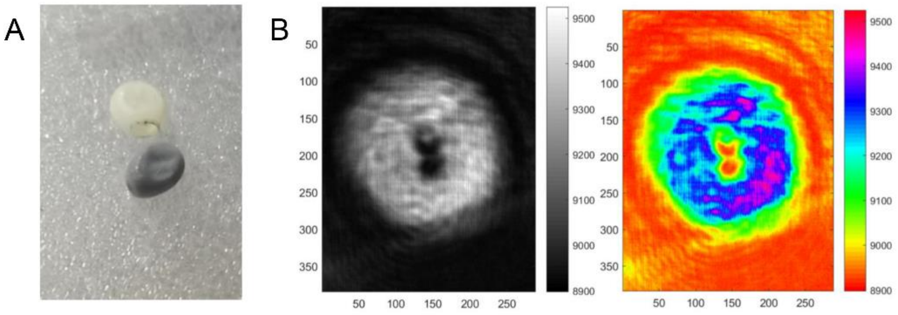

Figure S3 Comparison of imaging between silkworm shell and silkworm egg, Related to Figure 2. (A) Optical image. (B) THz images

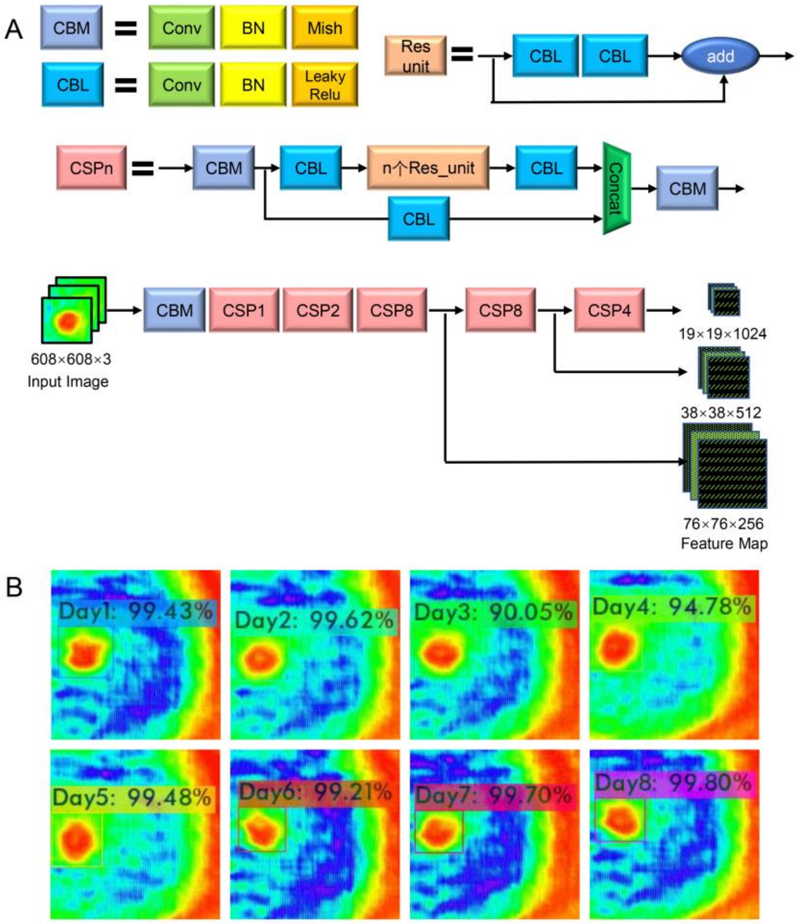

Figure S4. Evaluation of the model performance, Related to Figure 3. (A) DSPDarknet53 structure. (B) Intelligent THz recognition results of different development stages of a specific silkworm egg.

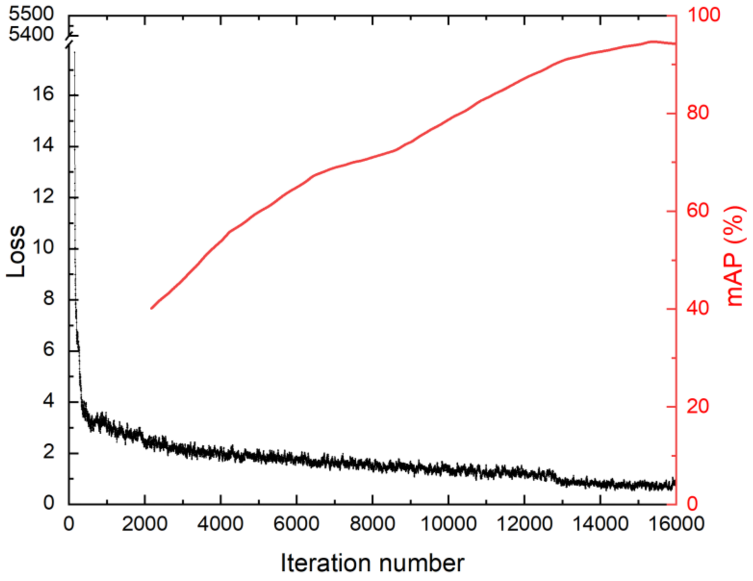

Figure S5 Training loss curves of the YOLO v4 silkworm egg recognition model before optimization, Related to Figure 4.

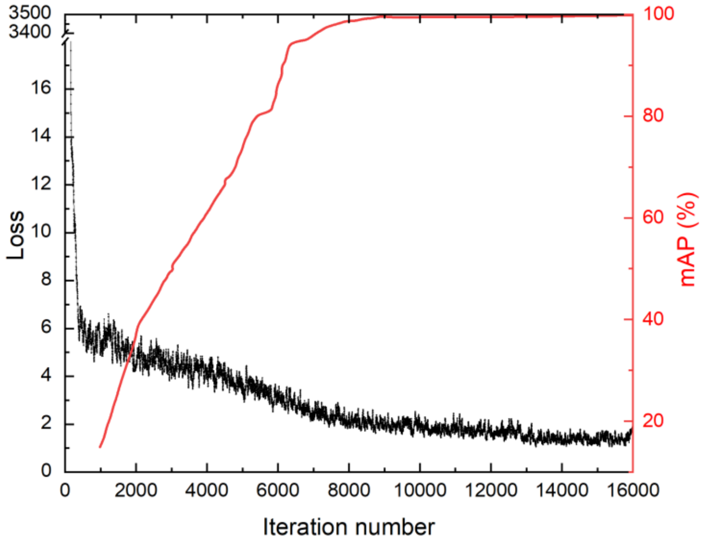

Figure S6 Training loss curves of the YOLO v4 silkworm egg recognition model after optimization, Related to Figure 4.

Table S1. Anchor box shape and average *IoU,* Related to STAR Methods.

	Anchor box	Avg *IoU*
1	80, 59/ 92, 56/ 80, 65/ 90, 63/ 85, 70/ 93, 71/ 102, 65/ 100, 75/ 112, 74	93.83%
2	78, 59/ 89, 57/ 83, 66/ 94, 62/ 92, 68/ 106, 65/ 92, 76/ 102, 71/ 109, 78	93.94%
3	80, 56/ 80, 63/ 92, 56/ 90, 63/ 85, 70/ 102, 64/ 95, 70/ 110, 70/ 101, 77	93.93%
4	79, 60/ 91, 56/ 91, 62/ 84, 68/ 94, 67/ 105, 65/ 99, 72/ 94, 78/ 112, 75	93.90%
5	78, 62/ 88, 56/ 87, 63/ 98, 60/ 95, 66/ 88, 72/ 99, 72/110, 68/ 104, 79	93.93%
